# Plasma Circulating Cell-Free DNA Facilitated the Detection of an Alveolar Echinococcosis Patient Initially Misdiagnosed as Cystic Echinococcosis: A Case Report

**DOI:** 10.3390/tropicalmed9040088

**Published:** 2024-04-19

**Authors:** Yanping Zhao, Yiyang Shi, Shu Shen, Yan Zhang, Gengfu Wei, Xin Jin

**Affiliations:** 1 BGI Research, Shenzhen 518083, China; zhaoyanping@genomics.cn (Y.Z.); zhangyan15@genomics.cn (Y.Z.); 2 Department of Hepatobiliary Surgery II, The People’s Hospital of Ganzi Tibetan Autonomous Prefecture, Kangding 626000, China; 19183736892@163.com; 3 Department of Liver Surgery, West China Hospital of Sichuan University, Chengdu 610041, China; shenshu9_9@163.com

**Keywords:** alveolar echinococcosis, cystic echinococcosis, cell-free DNA, cfDNA, misdiagnosis, *Echinococcus granulosus sensu lato*, *Echinococcus multilocularis*

## Abstract

Echinococcosis, especially alveolar echinococcosis (AE), is becoming an emerging/re-emerging disease with a growing number of cases reported globally. The diagnosis of echinococcosis is based mainly on imaging, which may be challenging when the image presentation is atypical. We reported one patient with suspected cystic echinococcosis (CE) by imaging. The cell-free DNA (cfDNA) obtained from sequencing the patient’s plasma before the operation showed that this patient probably had AE with 45 reads mapped to the *Echinococcus multilocularis* reference genome (Read-Pairs Per Million = 0.24). The patients underwent surgery, and the pathological result showed that the patient had AE. The conventional polymerase chain reaction (PCR) of her lesion sample extraction also indicated that the infection was caused by *Echinococcus multilocularis*. The follow-up ultrasound after three months indicated no recurrence. We demonstrated that the differentiation of CE and AE by imaging may not be that easy, with further elaboration on the differentiation between AE and CE in different aspects. We demonstrated that it is possible to use patients’ plasma cfDNA mapped to *Echinococcus* references before the operation to obtain the objective clue of the lesion to facilitate diagnosis.

## 1. Introduction

Over one million people are living with cystic echinococcosis (CE) globally [1]. Additionally, it is estimated that another 17,400 new alveolar echinococcosis (AE) cases occur annually, mostly in the Northern Hemisphere [2]. Increasing reports suggest that echinococcosis, especially AE, is becoming an emerging/re-emerging disease with growing reported cases globally [3,4,5,6,7]. As one of the 17 diseases targeted to be eliminated in the World Health Organization Neglected Tropical Diseases Roadmap, echinococcosis does not seem to be disappearing yet [8]. According to the World Health Organization (WHO) Foodborne Disease Burden Epidemiology Reference Group (FERG), echinococcosis causes an annual death of 19,300 persons and 871,000 disability-adjusted life-years (DALYs), and there is a cost of 3 billion US dollars for echinococcosis-related livestock loss and treatment annually [9]. 

Two main forms of echinococcosis affect humans, namely, CE, caused by *Echinococcus granulosus sensu lato*, and AE, caused by *Echinococcus multilocularis* [10]. A CE lesion is similar to a hepatic cyst, which can usually be surgically removed. AE is also known as “parasite cancer”, as it often grows in an aggressive manner, similar to cancer, invading important ducts inside and outside the liver, and leading to jaundice, portal hypertension, Budd-Chiari syndrome, and liver failure, with an over 90% mortality rate within 10 to 15 years if left untreated or inadequately treated [11]. As AE is prone to invade intra- and extra-hepatic ducts, the surgical procedure of AE is more complicated than that of CE. This complicated surgery often leads to an overall worse prognosis than that of CE. Furthermore, many AE patients go to hospitals too late and lose the opportunity to remove the lesions. Given the differences in the courses, management (especially surgical procedures), and prognosis and follow-up plans of CE and AE, it is very important to have an accurate diagnosis before surgical procedures [12].

According to the *Expert consensus for the diagnosis and treatment of cystic and alveolar echinococcosis in humans*, the diagnosis of echinococcosis mainly relies on clinical findings, imaging technology such as ultrasounds (US), conventional radiography (for thoracic and bone involvement), computed tomography (CT), and magnetic resonance imaging (MRI), and serology [13]; and it has been reported that misdiagnosis may occur when atypical features are presented in imaging [13,14,15,16]. As CE is more cosmopolitan, more doctors have experience dealing with CE; however, AE is also emerging in areas where no AE case was reported before [3,4,5,6,7,17]. In the endemic areas of western China, especially in the Tibetan Plateau region, AE is sometimes more prevalent than CE [18]. Many clinicians and imaging physicians do not have enough knowledge about and experience with AE because there are fewer chances to meet and manage AE patients [7,15,16]. Though serology could facilitate the differentiation between echinococcosis and other lesions in the liver most of the time, it could not differentiate between CE and AE; other limitations of serology include cross-reaction with other parasitic infections and false positivity in echinococcosis prevalent regions [11]. 

To facilitate the accurate diagnosis of human echinococcosis, different methods were developed. For example, Oberli, Chen, and Shang’s respective research teams developed multiplex polymerase chain reaction (PCR)-based methods that could differentiate between AE and CE using biopsy materials or tissue/lesion samples [15,19,20]. As not all echinococcosis patients would undergo surgery, tissue/lesion/biopsy materials are not always available. In recent years, more and more publications have indicated that the use of plasma cell-free DNA (cfDNA), especially *Echinococcus* cfDNA, could facilitate the diagnosis of echinococcosis [21,22,23]. 

We report here a case of echinococcosis that was originally diagnosed as CE by imaging technologies including CT, but later, the diagnosis was changed to AE according to pathological results. Interestingly, analysis of the patient’s plasma *Echinococcus* cfDNA results obtained through next-generation sequencing (NGS) also indicated that the patient had AE. 

## 2. Case Presentation

### 2.1. History

A 42-year-old woman presented to our hospital in Ganzi, Sichuan Province with a six-year history of echinococcosis in the liver. She was a Tibetan that had been living in the pastoral area of Ganzi County in Sichuan Province all her life. She was 155 cm in height and her weight was 69 kg; thus, her body mass index (BMI) was 28.72. She had felt pain in her upper abdomen six years earlier and went for a medical examination at the local hospital where she was diagnosed with echinococcosis. As the pain was not severe and occurred only occasionally, she did not pay much attention and no treatment was taken. In late 2021, she felt an expanding mass in her upper abdomen and came to seek treatment in our hospital on 10 October 2021. 

### 2.2. Investigations and Differential Diagnosis

**Figure 1:**
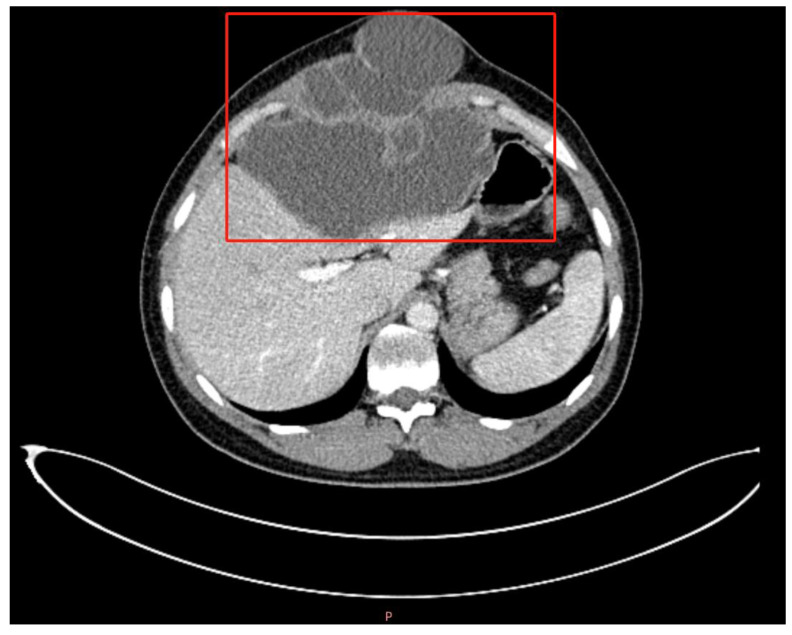
Abdominal CT image of the patient. The lesion was shown in the red box as a low-density cystic mass, with clear borders with the liver tissue. The “P” in the bottom means posterior.

CT imaging of the upper abdomen showed a well-defined cystic lesion region with multiple compartments, few calcifications, and the largest cross-section measuring 15.0 cm × 11.8 cm (Figure 1). The lesion region broke through the diaphragm to the anterior abdominal wall toward the skin and the cardio-diaphragmatic angle area. The left liver lobe was deformed by compression. The liver function was Child-Pugh A, and tumor markers including α-fetal protein, CA19-9, and CA12-5 were negative. No oedema, liver palms, or spider angioma was found. 

Most of her blood test indicators were in the normal range, except that the basophil percentage was slightly elevated (1.10%, reference range 0.00–1.00%). The results of the laboratory tests were as follows: total white blood cell (WBC) count, 7060/µL; eosinophilia, 200 cells/µL; basophil, 80 cells/µL (proportion of WBC, 1.10%); hemoglobin, 14 g/µL; total protein, 67.9 g/L; albumin, 37.1 g/L; total bilirubin, 5.9 μmol/L; aspartate aminotransferase (AST), 15 IU/L; alanine aminotransferase (ALT), 13 IU/L; alkaline phosphatase, 90 IU/L; gamma-glutamyl transferase, 28 IU/L; blood urea nitrogen, 5.9 mmol/L; and creatinine, 53.8 μmol/L. Serologic markers for viral hepatitis were negative. 

The clinical diagnosis based on imaging was hepatic cystic echinococcosis at a transitional stage of CE3. Preoperative diagnosis mainly relied on the imaging of CT scans. The hepatic cystic echinococcosis showed intrahepatic expansive growth. The CT showed cyst-like changes, due to an inflammatory reaction accompanied by the presence of thickened cystic walls, with clear boundaries with normal liver tissue. Early and transitional cystic density is also often homogeneous, while hepatic AE would usually show erosive growth in the CT, and the CT would show intrahepatic irregular shapes, tumor-like mixed density mass, and most often be accompanied by scattered calcifications.

### 2.3. The Plasma Cell-Free Echinococcus spp. DNA Analysis and ELISA Test

A blood sample of 5 mL was taken before the surgery to check the cell-free *Echinococcus* spp. DNA reads and for the anti-*Echinococcus* IgG test. The blood sample was first centrifuged at 1600× *g* at 4 °C for 10 min to obtain the plasma. After the centrifugation, 0.25 mL plasma was used for the lgG antibody test (Haitai Biotech, Inc., Zhuhai, China). Her anti-*Echinococcus* IgG test was positive. The rest of the plasma was then centrifuged again at 16,000× *g* at 4 °C for 10 min to remove cell debris as described before [21,23]. The plasma was sent to China National GeneBank (CNGB) in Shenzhen, Guangdong Province in a cold chain and was stored at −80 °C at CNGB for further cfDNA experiments. 

Plasma stored at −80 °C was thawed, and the QIAamp Circulating Nucleic Acid Kit (Qiagen, Hilden, Germany) was used to extract cfDNA from the plasma following the manufacturer’s manual. After extraction, cfDNA concentration was measured using a Qubit dsDNA HS Assay kit on a Qubit 2.0 Fluorometer (Invitrogen, Carlsbad, CA, USA) following the instructions. After the quality check, no fragmentation for the library preparation was taken as cfDNA was generally very short [21]. The qualified cfDNA was used to construct the sequencing library, and the library was sequenced using the DIPSEQ T1 platform (MGI, Shenzhen, China). A total of 208 million read pairs were obtained.

Previously validated cfDNA analysis workflow was used to analyze the cell-free *Echinococcus* spp. DNA reads [21]. In brief, raw data were first processed to remove low-quality reads and adaptors with SOAPnuke v2.1.0 (BGI-Shenzhen, Shenzhen, China) and Fastp v0.23.2 (HaploX Biotechnology, Shenzhen, China). Secondly, the clean data were mapped onto *Echinococcus* spp. sequence database with Kraken (University of Maryland, College Park, MD, USA). The *Echinococcus* spp. sequence database was constructed using *Echinococcus* spp. sequences downloaded from the NCBI GenBank database and chopped into 100 bp short pseudo-reads with a step size of 50 bp, then mapped to the possible host genomes to mask the possible host-contaminated pseudo-reads [21]. Thirdly, the mapped *Echinococcus* spp. DNA reads were mapped to the human reference genome to remove human cfDNA reads with Snap-aligner v1.0beta.23 (University of California, Berkeley, CA, USA) and low-complexity reads were removed with PRINSEQ (v0.20.4) (San Diego State University, San Diego, CA, USA). Finally, we removed those reads that were not best mapped to our constructed *Echinococcus* spp. sequence database (defined as identity < 97%, coverage < 92%, and e-value > 1 × 10^−5^) and those reads that mapped better to other taxa [21].

After the analysis, 49 *Echinococcus* spp. DNA reads were left, including 45 reads mapped to the *Echinococcus multilocularis* reference genome and another 4 reads mapped to the genus *Echinococcus* given the close relationship among the species in the genus *Echinococcus*. As most of the reads (91.84%) were mapped to the *Echinococcus multilocularis* reference genome, it was suggested that the patient was infected by *Echinococcus multilocularis*. Read-Pairs Per Million (RPM) was also calculated to standardize and estimate the concentration of *Echinococcus* spp. DNA. The RPM was defined as the *Echinococcus* spp. read counts per million sequencing data. The RPM of our patient was 0.24, using our analysis workflow [21,23]. 

### 2.4. The Treatment

The patient underwent surgery three days after the CT examination to remove the lesion. During the surgery, dense adhesion between the liver and diaphragm was observed. The cystic lesion invaded the IV segment of the liver and the round ligament of the liver, which grew forward to the upper abdominal wall and broke through the partial encroachments of the rectus muscle. Multiple cystic lesions were found in the abdomen as well. The abdominal wall of the inferior xiphoid and right upper abdominal diaphragm were invaded. The lesions and part of the invaded organs, including the liver, diaphragm, and abdominal wall, were resected and repaired. The operation time was 4 h, and the blood loss was 500 mL. No blood transfusion was made during the operation. Albendazole was prescribed later, a 600 mg bid for 25 consecutive days, and rest for 10 days, which should last for at least six months. She was advised to come back for a follow-up examination after three months. She indeed came back three months later and the ultrasound at her follow-up visit indicated no recurrence.

### 2.5. The Pathological Result

**Figure 2:**
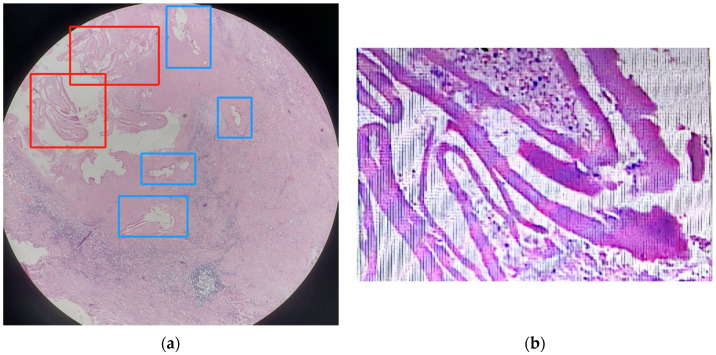
Microscopic images of the lesion through hematoxylin and eosin (H&E) staining. The (**a**) microscopic image (100×) of the liver including the lesion through H&E staining and the red boxes were two areas with central liquefied necrosis, and the four blue boxes were four pseudocysts; (**b**) enlarged microscopic image (400×) of the H&E staining of the central liquefied necrosis region.

The pathological result using the lesion sample (covered by liver tissue) showed that the lesion was probably hepatic alveolar echinococcosis, as it showed very obvious central liquefied necrosis regions (Figure 2). However, in contrast to what was described in the literature, the striation of the laminated layer was moderate rather than weak, and the color was pink instead of bluish-clear, as described in the literature for AE lesions in H&E staining [12]. 

### 2.6. The PCR Results

**Figure 3:**
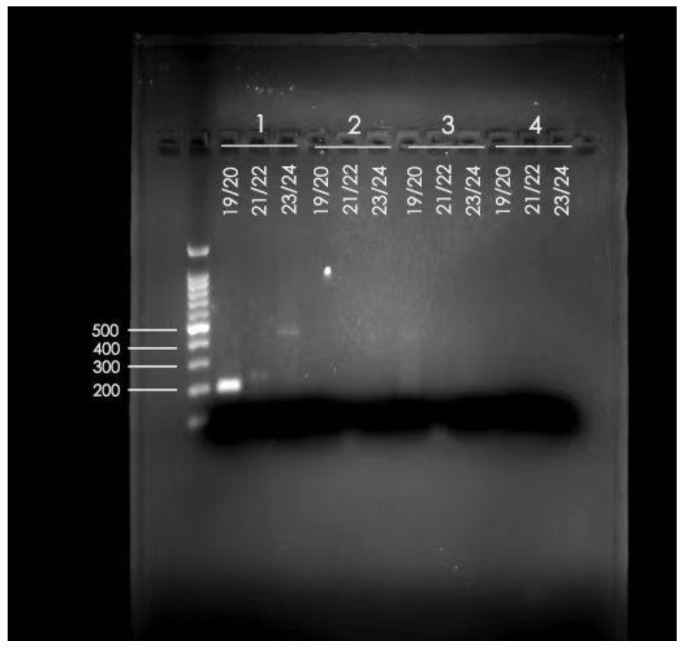
Gel electrophoresis of PCR products generated from the patient’s lesion sample extraction. 1 is the sample extraction from our patient; 19/20 was the primer for *Echinococcus multilocularis*; 21/22 was the primer for *Echinococcus granulosus sensu stricto*; 23/24 was the primer for *Echinococcus* species.

To verify the inconsistent results from the imaging, pathological results, and what was described in the literature, we further used conventional PCR of the lesion sample extraction to verify the accurate diagnosis, which indicated that the patient was indeed infected by *Echinococcus multilocularis* (Figure 3). Sample 1 on the left is the sample from our patient, and the primer pairs were using conventional PCR primers from the literature. Namely, 19/20 was the primer for *Echinococcus multilocularis* (Em-nest-for GTGAGTGATTCTTGTTAGGGGAAGA and Em-nest-rev ACAATACCATATTACAACAATATTCCTATC) [24]; 21/22 was the primer for *Echinococcus granulosus sensu stricto* (E.g.ss1-for. GTATTTTGTAAAGTTGTTCTA and E.g.ss1-rev. CTAAATCACATCATCTTACAAT) [25]; and 23/24 was the primer for *Echinococcus* species (Forward JB3 TTTTTTGGGCATCCTGAGGTTTAT and Reverse JB4.5 TAAAGAAAGAACATAATGAAAATG) [26]. These primers were used to amplify and detect the *Echinococcus* species using formalin-fixed paraffin-embedded tissue samples [27]. As shown in the gel electrophoresis, the tissue extraction PCR could map to 19/20 clearly and lightly to 23/24, confirming that the patient was infected by *Echinococcus multilocularis*. 

## 3. Discussion

Our patient here was diagnosed with CE before the operation and the staging was a transitional stage of CE3. However, her plasma cfDNA indicated that she was infected by the larvae of *Echinococcus multilocularis*; thus, the aetiological clue was different from the imaging result. 

### 3.1. The Difference between CE and AE

#### 3.1.1. The Difference between CE and AE in Aetiology and Staging Systems

In the aetiology, CE is caused by the infection of the larvae of *Echinococcus granulosus sensu lato*, and AE is caused by the larvae of *Echinococcus multilocularis* [10]. Compared to CE cases, AE infection is more aggressive and dangerous. *Echinococcus multilocularis* larva could spread to other organs either by infiltration or by metastasis, similar to cancer cells [13]. According to the WHO, based on the imaging findings, the AE stages could be classified into PNM staging, with P representing the parasitic mass in the liver, N representing the involvement of neighboring organs, and M representing metastases, while CE stages were classified as active (CE1 and CE2), transitional (CE3), and inactive (CE4 and 5) [13]. 

#### 3.1.2. The Difference between CE and AE in Geographical Distribution

There is also a difference between the geographical distribution of CE and AE [11]. In general, CE is more cosmopolitan than AE, covering nearly all lands on Earth with only a very small number of island countries announcing its elimination [28,29]. On the contrary, AE is more restricted in certain geographical regions, especially in the Northern Hemisphere [2]. A molecular investigation of near-complete/complete mitochondrial sequences of 92 patients’ samples from Tibet Autonomous Region (TAR) identified that the main *Echinococcus* species and genotypes infecting humans in TAR were *Echinococcus granulosus sensu stricto* G1 followed by G6 of *Echinococcus canadensis* (6.52%), and *Echinococcus multilocularis* (2.17%) [30]. However, in Shiqu County on the Tibetan Plateau of Sichuan Province, AE is more prevalent with a prevalence of 3.58%, which is significantly higher than the prevalence of CE at 2.31%, as reported in an ultrasound-based prevalence study screening on 84,742 residents [18]. 

Our patient is not from Shiqu County. Her hometown is Ganzi County, which borders Shiqu County. Furthermore, there are increasing reported AE cases in countries and regions without any cases of AE reported before [3,4,5,6,7,17]. As a result, doctors and hospitals should be better prepared and equipped to identify AE in the future. 

#### 3.1.3. The Difference between CE and AE in Imaging and Pathology

According to the *Expert consensus for the diagnosis and treatment of cystic and alveolar echinococcosis in humans*, 70% of AE cases presented with the following:

- “Juxtaposition of hyper and hypoechogenic areas in a pseudo-tumour with irregular limits and scattered calcification”;
- “Pseudo-cystic appearances due to a large area of central necrosis surrounded by an irregular hyperechogenic ring”;

And another 30% with atypical presentation such as haemangioma-like hyperechogenic nodules or a small, calcified lesion [13]. 

Our patient fits into none of these descriptions and was thus concluded as having CE according to imaging. 

For those lesions that meet the surgical requirement, the pathology result using the lesion that is taken out is more accurate. However, a lot of the patients would not go through surgery either because the lesion was too small, or because the patients were at a very late stage of the disease, especially with AE, and missed the opportunity to undergo surgery. 

In a summary of the pathologic presentation of 138 lesion samples from 112 echinococcosis patients (59 AE and 53 CE), it was summarized that six morphologic criteria could be used to differentiate CE and AE, including the following:

1. Size of the smallest cyst (CE/AE: >2/≤2 mm)
2. Size of the largest cyst (CE/AE: >25/≤25 mm)
3. Thickness of the laminated layer (CE/AE: >0.15/≤0.15 mm)
4. Peri-cystic fibrosis (CE/AE: >0.6/≤0.6 mm)
5. Striation of laminated layer (CE/AE: moderate-strong/weak)
6. Number of cysts (CE/AE: ≤9/>9) [12]

As these criteria were from the literature but not included in the diagnostic guidelines, they were not routinely recorded in clinical practice. The summary highlighted that in the H&E staining in pathology, the laminated layer of CE was moderate to strong, and the laminated layer of AE was rather weak in bluish [12]. The H&E staining from our case was not very typical for AE according to the description in the literature. However, based on our experience in dealing with echinococcosis before, the pathological diagnosis of AE was made. The rationale behind the judgement was as follows:

1. The size of the individual pseudocysts was smaller, usually between 1 mm and 2 cm in diameter.
2. The striation of the AE laminated layer was not very obvious.
3. Under microscopy, necrotic degeneration was often around the parasitic lesion, which is generally poorly demarcated from the surrounding tissue. CE, on the other hand, is generally a single cyst, larger in diameter, with an inner wall that is often smooth and demarcated from the surrounding area.

Probably due to transportation difficulty and mild or no symptoms, echinococcosis patients, especially AE patients in our hospital, often come to the hospital at a late stage, which could be the reason for the difference in the H&E presentation. As in the pathology paper, only 24 samples were molecularly confirmed as AE samples, and the setting of sample collection is in the University Hospital Zurich, which may not contain enough late-stage lesion samples [12]. 

### 3.2. Misdiagnosis of Echinococcosis

In a paper published in China in 2019, 140 echinococcosis patients’ lesion samples were included, and it was reported that the misdiagnosis rate between CE and AE was 6.4% (9/140), and another 8.6% (12/140) samples could not be classified into AE or CE. Furthermore, this paper reported that the misdiagnosis was higher in AE cases (12.9%, 4/31) than in CE cases (4.6%, 5/109); similarly, the unclassified cases happened more frequently in the AE group (16.1%, 5/31) than in the CE group (6.4%, 7/109) [16]. It was worth noticing that these 140 patients’ infections were confirmed by sequencing of PCR product after the amplification of the partial *COX1* gene of the genome DNA extracted from the ethanol-preserved tissues obtained from the surgical procedures [16]. This kind of sample could only be obtained after the surgery and could not be of reference to guide the surgical procedures. 

None of the 16 AE patients reported in Hungary received a preliminary diagnosis of AE, probably because it was believed to be an AE-free European country [7]. The final confirmation of AE was through histopathology and/or detection of *Echinococcus multilocularis* nucleic acid sequences. Out of the 16 AE patients, 11 were initially diagnosed as different tumors, such as liver tumor, hepatocellular carcinoma, liver metastasis, and adenocarcinoma; one was diagnosed as atypical hepatic cyst, four patients were suspected as echinococcosis, including one suspected of echinococcosis with no AE or CE differentiation, one suspected of either liver tumor or echinococcosis, two probably CE, but could also be metastasis, tumor, or haemangioma [7]. 

More complicated cases could be the co-infection of both *Echinococcus granulosus* and *Echinococcus multilocularis*. In one report from Qinghai Province, which is also a province heavily affected by both CE and AE, three co-infected cases were reported, out of which, only one was diagnosed corrected before surgery and the other two cases were both diagnosed as CE [31]. 

Our patient was not a co-infection according to pathology, PCR, and *Echinococcus* cfDNA results. She was diagnosed in our hospital, namely, the People’s Hospital of Ganzi Tibetan Autonomous Prefecture. Both the hospital and the doctors here have extensive experience dealing with both AE and CE patients, unlike regions such as Europe, where doctors might be less exposed to AE. Even experienced doctors could make a wrong judgment, so it might be a good idea to involve more objective measures when doctors are facing atypical image presentation. If she had been diagnosed with AE six years ago, she could have been followed up more regularly and the lesion could have been removed at an earlier stage.

### 3.3. Using cfDNA NGS to Facilitate the Diagnosis of Echinococcosis

A recent paper reported using the NGS method to facilitate the diagnosis of a suspected case of cerebral echinococcosis who had negative results for the known bacterial, tuberculosis, and fungal infections, and was then tested with NGS for his cerebrospinal fluid (CSF) [32]. This NGS method using a CSF sample facilitated the correct diagnosis. The use of NGS in plasma samples could be utilized to avoid misdiagnosis in echinococcosis, which is demonstrated in our case report. Though some *Echinococcus* cfDNA reads could not be grouped into specific species (4/49 in our case), the majority of the *Echinococcus* cfDNA reads could be classified into the correct species, and thus could be used to facilitate the diagnosis. The advantage of using plasma cfDNA to facilitate the diagnosis is that the result was molecular-based and more objective and could be obtained before the surgery, thus different from a lot of the other PCR-based molecular methods using tissue samples which could only be obtained after the surgery [15,19,20]. 

Though it was concluded in 2016 that *Echinococcus* cfDNA would not enter the blood circulation and could not be a candidate tool for echinococcosis diagnosis [33], this conclusion was updated in 2021, as increasing reports using sequencing methods to facilitate echinococcosis diagnosis were published [22]. Given that there are difficulties in some unclassified cases by imaging and the chances of misdiagnosis, the application of *Echinococcus* cfDNA could be a tool to facilitate the diagnosis in challenging cases as demonstrated by our case report. Having said that, to make the *Echinococcus* cfDNA test more affordable and accessible, further efforts on how to lower the cost should be investigated. 

## 4. Conclusions

It was often taken for granted that imaging patterns are different between AE and CE except in some cases of the “cystic lesion” (CL) stage, an undifferentiated stage [13]. We demonstrated here that there could be atypical presentations of the lesion in imaging and misdiagnosis could happen. Misdiagnosis is more often observed in AE cases. Objective measures could be utilized to facilitate the differentiation between AE and CE and other similar diseases through imaging methods with challenging cases. *Echinococcus* cfDNA of the suspected patient’s blood could facilitate the accurate differential diagnosis before surgery. More samples would be needed to validate the diagnostic performance. Further methods to reduce the cost should be explored to make the threshold of using this objective method in the management of echinococcosis.

## Data Availability

The data presented in this study are available on request from the corresponding authors. To ensure the participants’ privacy, the data are not publicly available.
